# Poor Mental Health and the Use of Buy Now, Pay Later Loans

**DOI:** 10.1001/jamahealthforum.2025.5620

**Published:** 2025-12-12

**Authors:** Hridika Shah, Emma Prus, Brian Castrucci, Sandro Galea, Catherine K. Ettman

**Affiliations:** 1 Department of Health Policy and Management, Johns Hopkins Bloomberg School of Public Health, Baltimore, Maryland; 2 de Beaumont Foundation, Bethesda, Maryland; 3 School of Public Health, Washington University, St Louis, Missouri; 4 Editor, *JAMA Health Forum*

## Abstract

This cross-sectional study examines the association between buy now, pay later loans and probable depression, anxiety, and posttraumatic stress.

## Introduction

Poor mental health is associated with financial precarity.^1^ A hidden cost of mental illness may be a predisposition to using financial tools that perpetuate financial precarity.

Buy now, pay later (BNPL) loans are short-term, interest-free debt that allow borrowers to pay for purchases in installments. The US Consumer Finance Protection Bureau indicates that the underregulated and risky business model of BNPL may encourage loan stacking (simultaneously taking out multiple loans across different vendors) and sustained use (habitual BNPL borrowing), reducing an individual’s ability to meet other financial obligations.^2^ We tested the association between probable depression, anxiety, and posttraumatic stress (PTS) and BNPL use.

## Methods

This study used data from wave 5 (collected from March to April 2024; analyzed from March to May 2025) of the nationally representative, longitudinal Cumulative Life Stressors Impact on Mental Health and Well-Being (CLIMB) study of US adults aged 18 years.^3^ The parent study was deemed exempt by the NORC institutional review board; Johns Hopkins Bloomberg School of Public Health deemed the deidentified secondary data analysis as not human participants research. The study adhered to STROBE reporting guidelines. We applied custom weights to align the sample with national benchmarks.

The main binary outcome was self-reported past-year BNPL use. The main binary exposures were probable depression, anxiety, and PTS.^4^ We calculated demographic characteristics of the sample by BNPL use and used multivariable logistic regression to calculate the odds of BNPL use by poor mental health, adjusting for age, gender, race and ethnicity, marital status, education, household income, and employment across 4 models (probable depression, anxiety, PTS, and all 3 indices). *P* values were 2-sided (*P* < .05). We used Stata, version 18.0 (StataCorp).

## Results

Table 1 shows the unweighted frequencies and weighted percentages of demographic characteristics, poor mental health, and BNPL use. Of 2121 participants, 341 (15.5%) used BNPL during the previous 12 months. Overall, being female, younger age, being unmarried, employment status, and probable depression, anxiety, and PTS were significantly associated with use of BNPL.

**Table 1.** Demographic Characteristics by Buy Now, Pay Later (BNPL) Use^a^

Characteristic	Use, No. (%)		Total (N = 2121)	*P* value^b^
	BNPL (n = 341 [15.5%])^c^^,^^d^	No BNPL (n = 1780 [84.5%])		
Age, y				
18-39	125 (39.6)	561 (36.1)	686 (36.7)	.003
40-59	129 (38.2)	547 (29.9)	676 (31.2)	
≥60	87 (22.2)	672 (33.9)	759 (32.1)	
Gender				
Female	196 (61.0)	879 (49.4)	1075 (51.2)	<.001
Male	145 (39.0)	901 (50.6)	1046 (48.8)	
Race and ethnicity				
Hispanic	70 (23.3)	259 (15.9)	329 (17.1)	.03
Non-Hispanic Black	42 (14.3)	164 (11.4)	206 (11.8)	
Non-Hispanic White	207 (54.3)	1236 (63.2)	1443 (61.8)	
Other^h^	22 (8.1)	121 (9.5)	143 (9.3)	
Marital status				
Not married	181 (57.5)	761 (48.0)	942 (49.5)	.01
Married	160 (42.5)	1019 (52.0)	1179 (50.5)	
Education				
Some college or less	231 (68.7)	1058 (63.5)	1289 (64.3)	.09
College graduate or more	110 (31.3)	722 (36.5)	832 (35.7)	
Income, $				
≤19 999	35 (14.6)	178 (12.1)	213 (12.5)	.20
20 000-49 999	102 (27.4)	413 (24.3)	515 (24.8)	
50 000-74 999	84 (22.6)	354 (19.9)	438 (20.3)	
≥75 000	120 (35.4)	835 (43.7)	955 (42.4)	
Employment				
Working full time	179 (49.3)	857 (45.2)	1036 (45.8)	<.001
Working part time	42 (13.8)	176 (10.4)	218 (10.9)	
Looking for work or unemployed	12 (4.9)	64 (5.3%)	76 (5.2%)	
Retired	41 (12.3)	445 (22.6)	486 (21.0)	
Homemaker, student, or other	20 (5.8)	131 (9.9)	151 (9.3)	
On leave (parental, sick, disability)	47 (13.9)	107 (6.6)	154 (7.8)	
Probable depression^e^				
No	233 (63.4)	1466 (79.3)	1699 (76.9)	<.001
Yes	108 (36.6)	310 (20.7)	418 (23.1)	
Probable anxiety^f^				
No	268 (73.5)	1549 (85.4)	1817 (83.6)	<.001
Yes	73 (26.5)	229 (14.6)	302 (16.4)	
Probable PTS^g^				
No	291 (81.0)	1642 (92.0)	1933 (90.3)	<.001
Yes	50 (19.0)	136 (8.0)	186 (9.7)	

Abbreviation: PTS, posttraumatic stress.

^a^ Estimates were derived from data from wave 5 of the Cumulative Life Stressors Impact on Mental Health and Well-Being study collected from March 27, 2024, to April 29, 2024, that were nationally representative of US adults 18 years and older. Among 3005 eligible individuals, 2144 (71.3%) completed the survey. We excluded 23 incomplete survey responses, yielding a final sample of 2121 participants. Missing data in the study included 2 participants with missing depression symptoms only, 2 participants with missing PTS symptoms only, and 2 participants with missing depression and anxiety symptoms.

^b^ Two-tailed χ^2^ analysis conducted for significance testing.

^c^ Any past 12-month use of BNPL services.

^d^ Frequencies are unweighted. Percentages are weighted. Categories may not add up to the total number due to missing data. Percentages may not add up to 100 due to rounding.

^e^ Probable depression defined as Patient Health Questionnaire–9 score of 10 or greater.

^f^ Probable anxiety defined as General Anxiety Disorder–7 score of 10 or greater.

^g^ Probable PTS defined as Primary Care PTSD Screen for *Diagnostic and Statistical Manual of Mental Disorders* (Fifth Edition) score of 3 or greater.

^h^ Included non-Hispanic Asian/Pacific Islander, multiracial, and other race.

Table 2 shows the results from the multivariable logistic regression models for the odds of BNPL use across the 4 models with probable poor mental health. Individuals with probable depression, anxiety, or PTS had odds ratios of 1.91 (95% CI, 1.28-2.84), 1.77 (95% CI, 1.20-2.61), and 2.35 (1.42-3.89), respectively, of using BNPL compared with individuals who did not have poor mental health. When including all 3 poor mental health indices in 1 model, only PTS remained significantly associated with BNPL use.

**Table 2.** Odds Ratio (OR) of Buy Now, Pay Later Use by Probable Poor Mental Health^a^^,^^b^^,^^c^^,^^d^

Characteristic	Model, OR (95% CI)			
	1: Depression	2: Anxiety	3: PTS	4: All 3 indices
Poor mental health				
Depression^e^	1.91 (1.28-2.84)	NA	NA	1.58 (0.98-2.53)
Anxiety^f^	NA	1.77 (1.20-2.61)	NA	1.14 (0.68-1.91)
PTS^g^	NA	NA	2.35 (1.42-3.89)	1.97 (1.19-3.27)
Reference	1 [Reference]	1 [Reference]	1 [Reference]	1 [Reference]
Age, y				
18-39	1.28 (0.78-2.10)	1.33 (0.81-2.19)	1.34 (0.84-2.15)	1.21 (0.74-1.98)
40-59	1.56 (0.97-2.51)	1.64 (1.03-2.61)	1.57 (0.98-2.54)	1.48 (0.93-2.36)
≥60	1 [Reference]	1 [Reference]	1 [Reference]	1 [Reference]
Gender				
Female	1.55 (1.13-2.12)	1.57 (1.15-2.14)	1.62 (1.21-2.19)	1.58 (1.16-2.15)
Male	1 [Reference]	1 [Reference]	1 [Reference]	1 [Reference]
Race and ethnicity				
Hispanic	1.53 (1.10-2.13)	1.49 (1.08-2.05)	1.48 (1.07-2.04)	1.51 (1.09-2.11)
Non-Hispanic Black	1.33 (0.77-2.31)	1.33 (0.77-2.31)	1.31 (0.76-2.25)	1.33 (0.76-2.32)
Non-Hispanic White	1 [Reference]	1 [Reference]	1 [Reference]	1 [Reference]
Other^h^	1.01 (0.60-1.70)	1.00 (0.60-1.69)	0.97 (0.57-1.66)	0.98 (0.58-1.66)
Marital status				
Not married	1.23 (0.90-1.68)	1.27 (0.94-1.73)	1.25 (0.92-1.70)	1.22 (0.89-1.67)
Married	1 [Reference]	1 [Reference]	1 [Reference]	1 [Reference]
Education				
Some college or less	1.05 (0.78-1.41)	1.10 (0.82-1.46)	1.14 (0.84-1.53)	1.08 (0.81-1.45)
College graduate or more	1 [Reference]	1 [Reference]	1 [Reference]	1 [Reference]
Household income, $				
≤19 999	0.89 (0.48-1.64)	0.87 (0.49-1.57)	0.90 (0.48-1.67)	0.85 (0.47-1.55)
20 000-49 999	1.19 (0.76-1.86)	1.21 (0.79-1.86)	1.17 (0.77-1.79)	1.16 (0.75-1.80)
50 000-74 999	1.21 (0.77-1.89)	1.23 (0.79-1.92)	1.17 (0.75-1.84)	1.16 (0.74-1.82)
≥75 000	1 [Reference]	1 [Reference]	1 [Reference]	1 [Reference]
Employment				
Working part time	1.05 (0.62-1.79)	1.05 (0.62-1.80)	1.01 (0.58-1.74)	1.00 (0.58-1.74)
Looking for work or unemployed	0.66 (0.30-1.45)	0.71 (0.34-1.46)	0.73 (0.35-1.53)	0.66 (0.31-1.40)
Retired	0.67 (0.41-1.11)	0.68 (0.42-1.10)	0.64 (0.40-1.05)	0.64 (0.39-1.04)
Homemaker, student, or other	0.44 (0.26-0.75)	0.46 (0.26-0.79)	0.45 (0.27-0.74)	0.43 (0.26-0.69)
On leave (parental, sick, disability)	1.64 (0.92-2.91)	1.72 (0.97-3.06)	1.69 (0.99-2.90)	1.48 (0.84-2.60)
Working full time	1 [Reference]	1 [Reference]	1 [Reference]	1 [Reference]

Abbreviations: NA, not applicable; PTS, posttraumatic stress.

^a^ Data from the Cumulative Life Stressors Impact on Mental Health and Well-Being study were collected from March 27, 2024, to April 29, 2024 (N = 2121). Complete case analysis used for multiple logistic regression resulting in 2117 participants in model 1, 2119 in models 2 and 3, and 2115 in model 4.

^b^ Missingness: 2 participants with missing depression symptoms only; 2 participants with missing PTS symptoms only; and 2 participants with missing depression and anxiety symptoms.

^c^ Controls: all models were adjusted for age, gender, race and ethnicity, marital status, education, household income, and employment.

^d^ Variable definitions: buy now, pay later service use defined as any past 12-month use.

^e^ Probable depression defined using the Patient Health Questionnaire–9 score of 10 or greater.

^f^ Probable anxiety defined using the General Anxiety Disorder–7 score of 10 or greater.

^g^ Probable PTS defined using the Primary Care PTSD Screen for *Diagnostic and Statistical Manual of Mental Disorders* (Fifth Edition) score of 3 or greater.

^h^ Included non-Hispanic Asian/Pacific Islander, multiracial, and other race.

## Discussion

This cross-sectional study found that after adjusting for demographic characteristics, probable depression, anxiety, and PTS were associated with greater odds of BNPL use. Our findings were consistent with previous work that suggested that people with poor mental health may experience challenges with financial decision-making.^1^ Due to its data-driven, hyperpersonalized user interface design, BNPL may generate a feedback loop of unplanned purchases.^2^ BNPL availability increases impulsive purchases by 13%.^5^ BNPL use also increases levels of unsecured consumer debt, which contributes to poorer health and foregone medical care.^6^ These risks may be amplified for people with poor mental health.

However, for individuals with credit constraints and smaller savings, BNPL may be a more appropriate tool than other risky forms of credit (ie, high-interest credit cards and payday loans).^2^ Moreover, the direction of BNPL use may go in either direction. That is, individuals with greater perceived needs for BNPL products may experience poor mental health because of their financial stressors.^1^

This study was limited by its cross-sectional analysis and lack of clinical diagnosis for measures for probable depression, anxiety, and PTS.^4^ The CLIMB measure of poor mental health used a past 2 weeks reference period, while BNPL use referred to any past-year use. Future work with longitudinal data may unpack these bidirectional associations. Notwithstanding these limitations, the study results suggest that probable poor mental health is associated with greater odds of BNPL use, underscoring the need for consumer protections to prevent deteriorating financial standing and worsening mental health among adults.

## Notes

Supplement. Data sharing statements
