# Supplementary material for: Poor Mental Health and the Use of Buy Now, Pay Later Loans
Source: JAMA Health Forum. 2025 Dec 12;6(12):e255620. doi: 10.1001/jamahealthforum.2025.5620 (PMC12701504; doi:10.1001/jamahealthforum.2025.5620)
Supplement: Supplement. — Data sharing statements [file jamahealthforum-e255620-s001.pdf]

Data Sharing Statement

Shah. Poor Mental Health and the Use of Buy Now, Pay Later Loans. *JAMA Health Forum*. Published December 12, 2025. doi:10.1001/jamahealthforum.2025.5620

Data
Data available: Yes
Data types: Data dictionary
How to access data: Data are available upon reasonable request for research purposes sent to CKE:cettman1@jhu.edu.
When available: With publication

Supporting Documents
Document types: None

Additional Information
Who can access the data: Data are available upon reasonable request for research purposes sent to CKE:cettman1@jhu.edu.
Types of analyses: Data are available upon reasonable request for research purposes sent to CKE:cettman1@jhu.edu.
Mechanisms of data availability: Data are available upon reasonable request for research purposes sent to CKE:cettman1@jhu.edu.
